# Access to the next wave of biologic therapies (Abatacept and Tocilizumab) for the treatment of rheumatoid arthritis in England and Wales: Addressing treatment outside the current NICE guidance

**DOI:** 10.1007/s10067-011-1936-6

**Published:** 2012-01-25

**Authors:** Yee Chiu, Andrew J. K. Ostor, Anthony Hammond, Katharina Sokoll, Marina Anderson, Maya Buch, Michael R. Ehrenstein, Patrick Gordon, Sophia Steer, Ian N. Bruce

**Affiliations:** 1 Department of Rheumatology, Wirral University Teaching Hospital, Wirral, UK; 2 Addenbrookes Hospital, Cambridge University Hospitals NHS Foundation Trust, Cambridge, Cambridgeshire UK; 3 Maidstone District General Hospital, Maidstone, Kent UK; 4 Bradford Teaching Hospitals NHS Foundation Trust, Bradford, West Yorkshire UK; 5 University Hospital Aintree, Clinical Sciences Centre, Liverpool, UK; 6 Chapel Allerton Hospital, Leeds, West Yorkshire UK; 7 University College London, Centre for Rheumatology, London, UK; 8 King’s College Hospital NHS Foundation Trust, Clinical and Academic Rheumatology, London, UK; 9 Arthritis Research UK Epidemiology Unit, Manchester Academic Health Sciences Centre, University of Manchester, Manchester, UK; 10 The Kellgren Centre for Rheumatology, Manchester NIHR Biomedical Research Centre, Central Manchester Foundation Trust, Manchester, UK

**Keywords:** Abatacept, Individual funding request, National Health Service, National Institute for Health and Clinical Excellence, Rheumatoid arthritis, Tocilizumab

## Abstract

Patients in England and Wales with rheumatoid arthritis (RA) receive treatment from the National Health Service (NHS) with therapies approved by the European Medicines Agency (EMA), under guidance from the National Institute for Health and Clinical Excellence (NICE). This document overviews the current NICE guidelines for the treatment of RA and identifies scenarios when such guidance may not represent the optimum management strategy for individual patients. Specifically, we consider the use of tocilizumab or abatacept as the most appropriate treatments for some patients. In such scenarios, it may be possible for the clinician to secure access to the required therapy through an application procedure known as an ‘individual funding request’, the process of which is described in detail here. At present, it is unclear the extent to which the proposed reform of the NHS will affect the role of NICE in providing guidance and setting standards of care. Until the full impact of the proposed changes are realized, individual funding requests will remain a valuable way of securing the optimal treatment for all patients suffering from RA.

**Electronic supplementary material:**

The online version of this article (doi:10.1007/s10067-011-1936-6) contains supplementary material, which is available to authorized users.

## Accessing biologics for rheumatoid arthritis (RA) in England and Wales

### National Institute for Health and Clinical Excellence (NICE)

NICE provides guidance, sets quality standards and manages a database to improve health and prevent and treat illness. Currently, there are ~580,000 people with RA in England and Wales (~1% of the population) [1]. The treatment goal for this chronic disease is to rapidly induce remission by suppressing inflammation and, thus, preventing joint destruction, loss of function and disability. For the patient, this translates into control of pain, maintenance of function and improved quality of life [1].

**Fig. 1:**
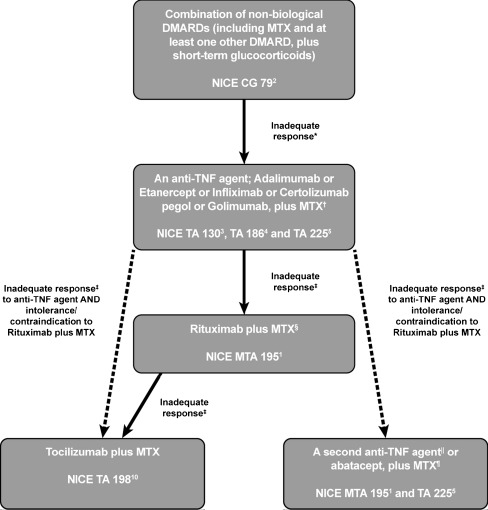
NICE guidance on the treatment of patients with rheumatoid arthritis. *Disease Activity Score 28 > 5.1 confirmed on ≥2 occasions, 1 month apart. ^†^If the patient is intolerant of MTX or if MTX is considered inappropriate, then adalimumab, etanercept or certolizumab pegol (but not infliximab or golimumab) may be given as monotherapy. ^‡^An adequate response is defined as an improvement in DAS28 of ≥1.2. ^§^Administered no more frequently than every 6 months; ^װ^Adalimumab, etanercept, infliximab or golimumab — there is currently no guidance for use of certolizumab pegol as a second anti-TNF agent. ^¶^If the patient is intolerant of MTX or if MTX is considered inappropriate, then adalimumab or etanercept may be given as monotherapy. *CG* clinical guideline, *TA* technology appraisal, *MTA* multiple technology appraisal

NICE have issued overarching guidelines for the management of RA which address diagnosis, pharmacological treatment and disease monitoring [2]. To supplement these guidelines, NICE takes European Medicines Agency (EMA)-approved therapies and performs technology appraisals (TA) to provide guidance on their cost-effectiveness and use within the National Health Service (NHS). The outcomes of these appraisals and how they influence access to therapy are overviewed (Fig. 1).

According to NICE guidelines, first-line treatment for RA should constitute a proactive approach, with early introduction of a combination of disease-modifying antirheumatic drugs (DMARDs) [2]. However, not all patients achieve an adequate response to non-biologic DMARDs and drug-related side effects may occur, limiting their use. Technology appraisals issued by NICE for the tumour necrosis factor α (TNF) inhibitors, adalimumab, etanercept, infliximab, certolizumab pegol and golimumab recommend the use of these biologic DMARDs, plus methotrexate (MTX), for the treatment of active disease [3–5]. If MTX use is contraindicated, adalimumab, etanercept or certolizumab monotherapy may be given [3, 4].

However, up to one-third of patients do not respond adequately or lose response to anti-TNFs over time, while some therapies are poorly tolerated [6]. A multiple technology appraisal (MTA195) [1] issued by NICE compared the clinical benefits and cost-effectiveness of treatment with a second anti-TNF, the B-cell depleting anti-CD20 monoclonal antibody, rituximab, or the T-cell co-stimulation modulator, abatacept, in patients refractory to, or intolerant of, an initial anti-TNF. Based on clinical and cost benefits, MTA195 recommends initial treatment with rituximab, plus MTX (if tolerated) every 6 months [1].

In cases of MTX intolerance, NICE recommends adalimumab or etanercept monotherapy [1]. For rituximab intolerance/contraindication, a second anti-TNF or abatacept, (+MTX), is recommended [1, 5]. Evidence suggests that, for some patients, switching to a treatment with a different mechanism of action may be more effective than switching within the same therapy class [7]. Furthermore, studies show that among patients who discontinue an anti-TNF due to efficacy or safety/tolerability, the same reason for discontinuation of a second anti-TNF will likely be reported [7, 8]. In addition, with the increased prevalence of tuberculosis (TB) in the UK, anti-TNFs may not be the most appropriate choice for certain high-risk patients [9].

The interleukin-6 receptor inhibitor, tocilizumab, did not have data available for inclusion in MTA195. However, TA198 recommends tocilizumab (+MTX), in patients refractory to an anti-TNF and rituximab (+MTX), or when rituximab is contraindicated/not tolerated in anti-TNF inadequate responders [10]. NICE offers no decision-making guidance for when to adhere to MTA195, or TA198; the decision often relies on the rheumatologist’s experience and patient’s preference.

### Gaps in NICE guidance

Based on cost-effectiveness, NICE recommends rituximab plus MTX, in anti-TNF inadequate responders [1]. However, studies suggest that not all patients benefit adequately from treatment. In a trial of 311 rituximab- (+MTX) treated patients who had failed a previous anti-TNF, 49% did not meet the primary endpoint (20% improvement in American College of Rheumatology criteria) [11]. Furthermore, subanalyses of clinical and registry data [11–16] demonstrate that rituximab is suboptimal in patients negative for rheumatoid factor and/or anti-cyclic citrullinated peptide. Since up to 20% of patients may be seronegative [11, 12, 16], therapies demonstrating efficacy in such patients, such as abatacept or tocilizumab, may be preferable. The consensus statement on rituximab use [17] supports consideration of alternative treatment in seronegative patients.

Rituximab has been reported to be well-tolerated in a study of 2,578 patients with 5,013 patient-years of exposure [18]; over five courses of treatment, serious adverse events did not increase over time. However, with repeated cycles (over 7 years), a decrease in immunoglobulins (IgA, IgM and IgG) has been observed [19], with low IgG levels associated with increased risk of serious infections [18]. In patients with risk factors for infection (age, glucocorticoid use), IgG levels should be monitored and alternative treatment considered, where appropriate [20]. Safety concerns for rare events, such as progressive multifocal leukoencephalopathy, have also been reported with rituximab use [21].

There is some concern that initiating another biologic before B-cell counts normalize may lead to an increased infection risk; a small study (*n* = 185) has suggested this is not the case [22]. However, this study had a relatively short follow-up (mean = 12 months). To our knowledge, there are no large-scale controlled clinical data on the time frame for safely switching patients from rituximab to another biologic; guidance informed by clinical data is required.

The next section overviews the evidence supporting tocilizumab and abatacept use (in-line with EMA approval) as equivalent treatment options to rituximab in anti-TNF inadequate responders, with the aim of facilitating decision-making on appropriate treatment for individual patients.

## Evidence for therapies outside NICE guidance: tocilizumab and abatacept as second-line biologics

Tocilizumab (+MTX or monotherapy) and abatacept (+MTX) are EMA-approved for use in patients who have failed conventional therapy (MTX) or anti-TNF therapy, based on evidence that treatment can slow damage to joints and improve physical function. The Scottish Medicines Consortium (SMC) recommends tocilizumab under these circumstances. In the US, tocilizumab (+MTX or monotherapy) is indicated in anti-TNF inadequate responders and abatacept (+MTX or monotherapy) in biologic-naïve patients.

Substantial evidence exists supporting tocilizumab and abatacept as equivalent options to rituximab in anti-TNF or MTX-inadequate responders, as approved in Europe and the US. A review of the efficacy and safety of both therapies in different populations is available as online supplementary material. There are also numerous systematic reviews providing comparisons of the relative efficacy and safety of biologics. For example, according to the European League Against Rheumatism (EULAR) Task Force [23], there is good evidence for the efficacy of abatacept in MTX-naïve patients and for the efficacy of abatacept and tocilizumab in both DMARD- and anti-TNF-inadequate responders. An increased risk of bacterial infection and TB with anti-TNF agents (compared with conventional DMARDs) was noted. Cochrane reviews using indirect comparisons of biologics in different patient populations (DMARD- and anti-TNF inadequate responders) support these findings, reporting similar efficacy for all biologics, with the exception of anakinra which appears less efficacious [24]. With regards to safety, abatacept was reported to be significantly less likely than infliximab, tocilizumab and certolizumab pegol to be associated with serious infections, while abatacept, adalimumab, etanercept and golimumab appeared significantly less likely than infliximab to result in withdrawals due to AEs [25].

## The proposed NHS reform

Although the Department of Health’s White Paper ‘Equity and Excellence: Liberating the NHS’ will no doubt result in significant changes in the processes related to patient care, it is unclear, at present, to what extent this will affect the role of NICE and in rheumatologists accessing the therapies required by their patients. One concern related to the NHS reform is that it may result in a ‘postcode lottery’, in which there is wide regional variation in treatment standards across England and Wales. In order to ensure this does not occur, a consensus unified approach on the best practice management of patients with RA is required.

A NICE-adapted algorithm detailing the successive use of biologics to facilitate the use of non-NICE approved therapies in certain scenarios has been developed by the Leeds Rheumatology Unit, with the intention of rolling-out for adoption in the Yorkshire region [26]. Such an algorithm is an example of how local guidelines can supplement national NICE guidance to help streamline patient management.

## Current procedures for obtaining funding

As previously detailed, different clinical scenarios exist for which current NICE guidance may prevent patients with RA accessing the most appropriate therapy for their clinical circumstances. In such scenarios, there are alternative means of securing treatment, for example, private funding through medical insurance or entering a clinical trial. Alternatively, an ‘individual funding request’ may be submitted to the governing PCT in cases where the consultant considers a patient’s case to be exceptional, and thus outside the NICE guidance.

### Individual funding requests

**Fig. 2:**
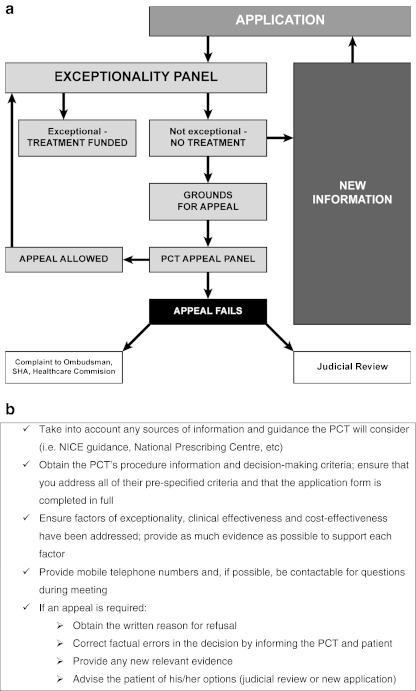
Individual funding requests. **a** General overview of the individual funding request process, reproduced by kind permission of the author [28]. **b** Checklist for applying for individual funding requests, adapted from ‘Your patient’s right to treatment’ [29]. *SHA* Strategic Health Authority

The general process of applying for an individual funding request is outlined in Fig. 2a (note that details may vary across regions).

An individual funding request application is assessed by a panel set up by the governing PCT. This panel is obliged to consider the patient’s application in conjunction with NICE guidance and any other applicable material, such as, local PCT criteria, information from the National Prescribing Centre and Centre for Evidence-based Purchasing, and relevant guidance from the SMC or the All-Wales Medicines Strategy Group (AWMSG). A typical submission consists of an application form provided by the PCT and a letter of support from the requesting consultant. The procedure information and decision-making criteria of the particular PCT should be carefully considered before making an application. A checklist for the rheumatologist in applying for individual funding requests is provided in Fig. 2b, and outlined in more detail in the next section.

## Decision-making criteria

The supporting letter from the requesting consultant should effectively illustrate three main points:

- Exceptionality — the patient is exceptional or exceptional circumstances are involved.
- Clinical effectiveness — the patient is ‘more likely than not’ to respond.
- Cost-effectiveness — the treatment is cost-effective for the particular patient.

### Exceptionality

Guidelines for determining exceptionality vary across PCTs. Broadly, exceptionality can be defined as ‘not usual’; it does not necessarily mean something extremely rare, unique or unprecedented. The fact that similar cases exist should not render the patient in question as ‘not exceptional’. Non-health aspects (such as age, employment status or other social factors) can also contribute to the exceptionality. Article 8 of the European Convention on Human Rights Act 1998, states that ‘everyone has the right to respect for his/her private and family life’. Therefore, a patient with close family members (who are either dependent on the patient, or carers of the patient), may have a greater chance of being exceptional, within the law, than a patient with no family members.

### Clinical effectiveness

An informative, concise patient history of the inadequate responses observed with prior treatments should be detailed. Appropriate research, data or publications relating to the clinical effectiveness of the requested treatment should be provided. If the patient has previously responded to treatment (through private funding or a clinical trial), this can be detailed. Finally, written support from a colleague that the requested therapy is the most appropriate treatment for the patient can strengthen an application.

### Cost-effectiveness

Often, PCTs will have limited cost-effectiveness data; providing a brief comparison of all predicted costs that will be incurred with/without treatment (hospital admissions, outpatient appointments, supplementary drugs), may be the deciding factor in a successful application. Most PCTs will not consider indirect costs (i.e. costs associated with time off work due to illness) when determining cost-effectiveness.

## The role of NICE

It is a point of law that NICE publishes guidance, not policy. Indeed, NICE states that their recommendations do not replace the knowledge and skills of health professionals responsible for making treatment decisions. In support of this, the Department of Health states that lack of NICE guidance is not an adequate reason to refuse funding [27].

Furthermore, NICE does not cover every eventuality, and the heterogeneity of RA may mean that standard processes are not applicable. When applying for funding, one should highlight how the specific case is unique, and thus outside the scope of NICE guidance. Similarly, any changes in the evidence base since publication of NICE guidance should also be taken into account by the PCT. This can be of particular importance as the majority of NICE guidance is not updated on an annual basis.

## Appeal process

An unsuccessful individual funding request can be appealed if; procedure was not followed (i.e. moving the meeting date without informing the applicant) or if the outcome was unlawful (i.e. refusing a NICE-approved treatment) or irrational (i.e. misuse or omission of relevant facts) [28, 29]. New evidence, such as proven benefit following private treatment, or a change in the patient’s circumstances that add to their exceptionality, should be presented to the appeal panel.

If the appeal fails, applicants can: submit a new application (appropriate in light of new evidence); file an official complaint (lengthy procedure unlikely to result in success); or initiate judicial review in the High Court (will only be successful if the PCT did not follow due process and thus acted unlawfully – not concerned with merits of individual cases).

## The road ahead

**Fig. 3:**
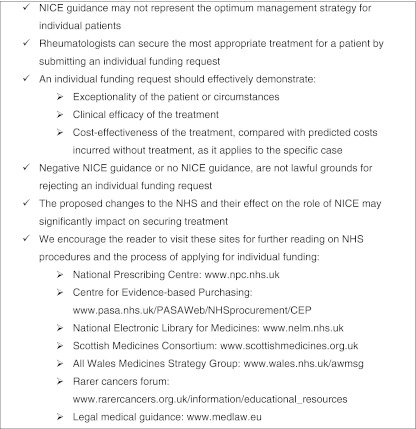
Key points: obtaining the optimal treatment for your patient

The key points discussed in this paper regarding access to therapy are summarized in Fig. 3, There are still a number of issues open to debate regarding obtaining treatment required by the considerable number of patients with RA in England and Wales: Does the availability of funding and the current process of requesting individual funding prevent or delay patients receiving the treatment they need? Should a standardized process for individual funding requests be implemented across England and Wales? Should there be a patient’s charter for applications and appeals within a set timeframe? Most importantly, what will be the impact of the proposed changes to the NHS and NICE on securing treatments both nationally and on a local level?

While effective EMA-approved treatments are available, the field of biologic therapy for RA is expanding rapidly, providing choice and hope for many patients. Placing the appropriate therapy, tailored to each individual, is based upon good evidence and clinical judgement. Access to these drugs is crucial to enable good management of RA; as such, rheumatologists should continue to act as strong advocates for their patients.

## Electronic supplementary material

Below is the link to the electronic supplementary material.

ESM 1 (DOC 530 kb)
