# Supplementary material for: Access to the next wave of biologic therapies (Abatacept and Tocilizumab) for the treatment of rheumatoid arthritis in England and Wales: Addressing treatment outside the current NICE guidance
Source: Clin Rheumatol. 2012 Jan 25;31(6):1005–12. doi: 10.1007/s10067-011-1936-6 (PMC3362712; doi:10.1007/s10067-011-1936-6)
Supplement: Supplementary file 1 — (DOC 530 kb) [file 10067_2011_1936_MOESM1_ESM.doc]

***ONLINE SUPPLEMENTARY TEXT***

*Tocilizumab*

The Phase III RADIATE (Research on Actemra Determining effIcacy after Anti-TNF failurEs) trial (which included 499 patients) supports use of tocilizumab plus MTX in anti-TNF inadequate responders [1]. The primary endpoint of an ACR 20 response at Week 24 was achieved by significantly (p<0.001) more patients receiving either 8 or 4 mg/kg tocilizumab plus MTX (50.0 and 30.4%, respectively) than placebo plus MTX (10.1%; Online Supplementary Figure 1). Similar improvements were observed for ACR 50 and 70 and DAS28. Patients responded regardless of the number of failed anti-TNFs, or the most recent agent failed [1].

**[Online Supplementary Figure 1]**

Tocilizumab plus MTX was well tolerated, with most adverse events (AEs) being mild-to-moderate [1]. The frequency of SAEs was higher (11.3%) in the placebo plus MTX group than in the 8 and 4 mg/kg tocilizumab plus MTX group (6.3 and 7.4%, respectively), primarily driven by complications of RA and infections. The rate of serious infections per 100 patient–years was 9.98, 5.72 and 9.64 in the 8 mg/kg, 4 mg/kg and placebo groups, respectively, resulting in four patients (two receiving tocilizumab and two receiving placebo, plus MTX) discontinuing. No deaths, incidences of tuberculosis or opportunistic infections were reported [1].

There is also evidence demonstrating short-term efficacy and safety of tocilizumab in patients with an inadequate response to MTX [2-4]. In the Phase II CHARISMA (Chugai Humanized Anti-Human Recombinant Interleukin-Six Monoclonal Antibody) [3] and Phase III OPTION (tOcilizumab Pivotal Trial in methotrexate Inadequate respONders) [4] studies, significantly more patients randomized to tocilizumab plus MTX achieved the primary endpoint of an ACR 20 score by Week 16 or Week 24, respectively, compared with placebo plus MTX patients. In a similar patient population, significant inhibition of radiographic progression and improvement in physical function were observed with tocilizumab plus MTX treatment in the Phase III LITHE (TociLIzumab Safety and THE Prevention of Structural Joint Damage) study [2,4]. Similar improvements in physical function outcomes, in addition to improvements in the signs and symptoms of RA and in disease activity, were observed in the Phase III TOWARD (Tocilizumab in COmbination With Traditional DMARD Therapy) study of patients with a previous inadequate response to at least one traditional DMARD [5]. Tocilizumab plus MTX was well tolerated, with a consistent safety profile throughout these trials.

The Phase III AMBITION (Actemra versus Methotrexate double-Blind Investigative Trial In mONotherapy) study investigated tocilizumab monotherapy in patients who had not failed previous treatment with MTX or biologics [6]. Although the primary endpoint of an ACR 20 response at Week 24 was achieved by a greater proportion of tocilizumab-treated patients than MTX-treated patients, tocilizumab resulted in a higher frequency of some safety events, including Grade 3 neutropaenia, increased total cholesterol and low-density lipoprotein cholesterol (3.1 vs 0.4%, 13.2 vs 0.4% and 3.1 vs 0% for tocilizumab vs placebo, respectively). The long-term clinical significance of these findings requires further investigation.

*Abatacept*

The efficacy and safety of abatacept has been examined in several clinical trials. The Phase III ATTAIN (Abatacept Trial in Treatment of Anti-TNF INadequate responders) study was the first randomized and controlled clinical trial to demonstrate the safety and efficacy of abatacept, plus DMARD, in patients with RA and an inadequate response to anti-TNFs [7]. Following a washout period of current anti-TNF, patients received abatacept (n=258) or placebo (n=133), plus DMARD. At Month 6, the co-primary endpoints were achieved by significantly more patients receiving abatacept versus placebo; 50.4 versus 19.5% (p<0.001), respectively, for ACR 20 response and 47.3 versus 23.3% (p>0.001), respectively, for a clinically meaningful change in physical function (Online Supplementary Figure 2). Secondary endpoints, such as the ACR 50 and 70, disease activity and health-related quality of life (HRQoL), were also significantly improved in the abatacept versus the placebo groups at Month 6 [7].

**[Online Supplementary Figure 2]**

Abatacept plus DMARD was well tolerated; AEs and SAEs occurred in 79.5 and 10.5% in the abatacept group, respectively, and 71.4 and 11.3% in the placebo group, respectively [7]. One death occurred; myocardial infarction and congestive heart failure in an abatacept-treated patient. Although infections were more common in the abatacept than the placebo group (37.6 versus 32.3% [p=0.30]), there were no unusual or opportunistic infections. Discontinuations due to infections were similar between groups (0.8 and 1.5% in the abatacept versus placebo group, respectively [p=0.61]) with serious infections reported in 2.3% of patients in each group. The most common infections were nasopharyngitis, sinusitis, upper respiratory tract infection and bronchitis.

Data from the long-term extension of ATTAIN demonstrated that the efficacy benefits observed at 6 months are sustained over 5 years of treatment, with a consistent safety profile [8]. The 6-month ARRIVE (Abatacept Researched in Rheumatoid arthritis patients with an Inadequate anti-TNF response to Validate Effectiveness) study supports the findings of ATTAIN, and demonstrates the safety of directly switching anti-TNF inadequate responders to abatacept without a washout period, an approach which may be more clinically relevant for daily practice [9]. Indeed, owing to the inclusion/exclusion criteria, the patient population in ARRIVE is considered representative of clinical practice; patients had high baseline disease activity and an inadequate response to up to three anti-TNFs (which they could have failed for efficacy or safety reasons). Abatacept could be administered as monotherapy (US only) and there were no limitations on the type of background DMARD. In addition, patients with a positive purified protein-derivative test were included.

*Post-hoc* analyses from ATTAIN and ARRIVE demonstrate that abatacept is efficacious regardless of the number of prior anti-TNFs failed or the reason for failure [9,10]. However, patients who fail ≥1 anti-TNF are likely to have more refractory disease. In a *post-hoc* analysis of ATTAIN, numerically more patients who had previously failed one anti-TNF achieved DAS28-defined remission or low disease activity at Year 1 (15.0 and 25.5%, respectively) than those who failed two (10.8 and 21.6%, respectively) [10]. Similarly, in ARRIVE, a good EULAR response was achieved by 29 and 21% of patients, respectively, who had previously failed one or three anti-TNFs [11].

Safety and efficacy of abatacept in patients with an inadequate response to a non-biologic DMARD was first established in the AIM (Abatacept in Inadequate responders to Methotrexate) study [12]. At Year 1, abatacept plus MTX significantly improved physical function, slowed the progression of structural joint damage and demonstrated improvements in the signs and symptoms of RA, compared with placebo plus MTX [12]. The longest assessment of abatacept treatment in MTX-inadequate responders to date demonstrates sustained efficacy and consistent safety over 7 years of treatment [13]. Integrated analysis of safety data from eight abatacept trials has demonstrated that the types and incidence rates of AEs, SAEs and events of special interest (infections, malignancies, autoimmune events) did not increase in the long-term versus the short-term period, nor did annual incidence rates increase with increased duration of exposure, suggesting a stable long term safety profile of abatacept [14]. These observations are supported by registry data, which are important in assessing long-term safety of treatment in real-world clinical settings [15].

An indirect comparison of abatacept with infliximab, in MTX inadequate responders was assessed in the Phase IIIb ATTEST (Abatacept or infliximab versus placebo, a Trial for Tolerability, Efficacy and Safety in Treating rheumatoid arthritis) study [16], in which patients received background MTX. At Month 6, both therapies demonstrated similar efficacy benefits and acceptable safety. Between Month 6 and Year 1, patients receiving abatacept demonstrated greater improvements in disease activity, signs and symptoms of RA and HrQoL, than patients receiving infliximab. However, ATTEST was not powered to compare differences between the active treatment arms [16]. Subsequent to Year 1, patients could enter the long-term extension to receive abatacept plus MTX. Patients randomized to abatacept maintained or increased their response during Year 2 [17]. Patients switching from infliximab to abatacept at Year 1 demonstrated improvements in efficacy, comparable with the original abatacept group by the end of Year 2 [17].

**Online Supplementary Figure 1: Efficacy of tocilizumab (RADIATE study) [1]**

**
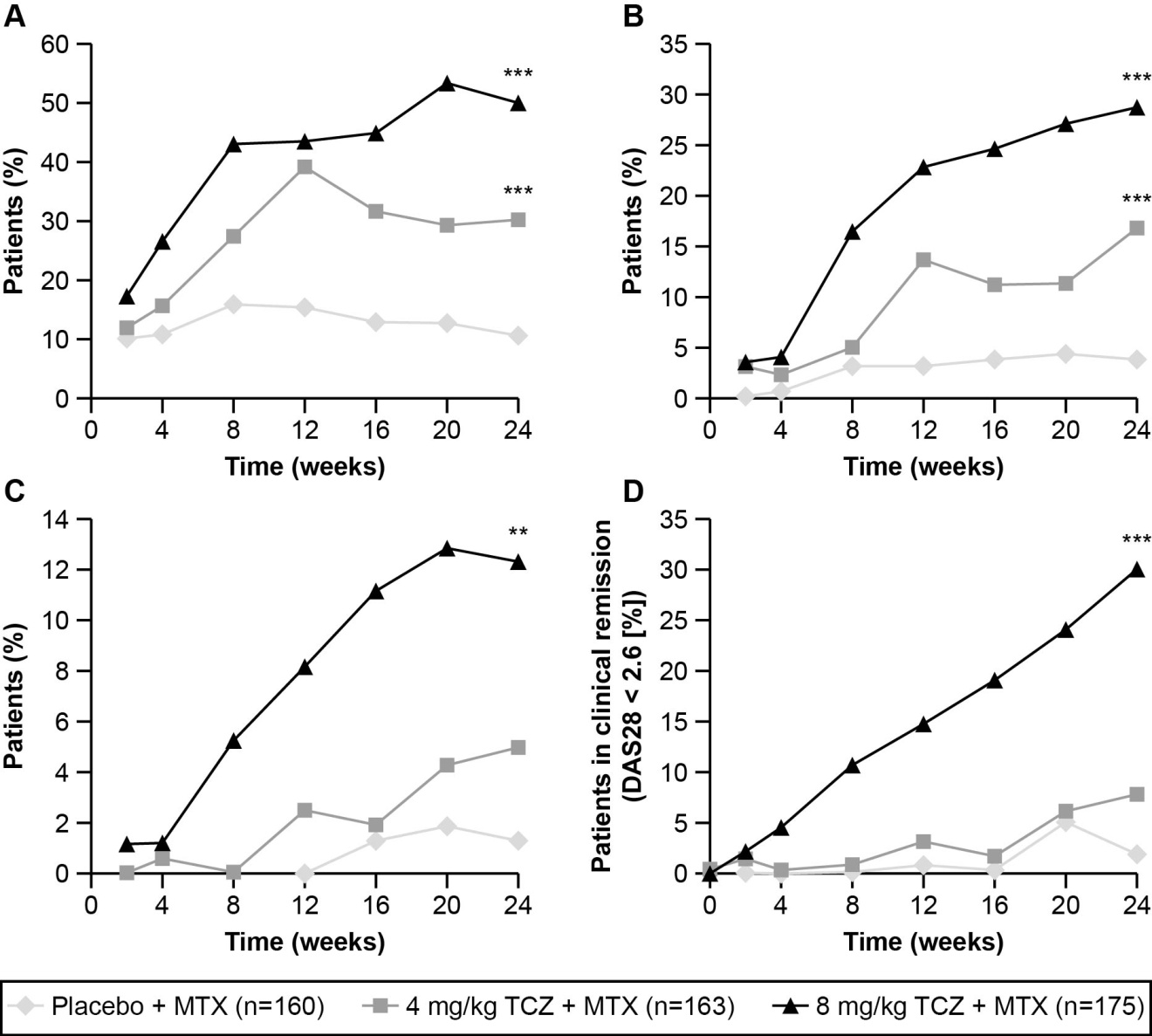
**

Clinical response to tocilizumab treatment by visit for (A) ACR20 response, (B) ACR50 response, (C) ACR70 response, (D) percentage of patients achieving clinical remission

(DAS28<2.6); **p<0.001 versus placebo; ***less than p<0.001 versus placebo; MTX=methotrexate; TCZ=tocilizumab; ACR=American College of Rheumatology; DAS28=Disease Activity Score 28. Reproduced from [17] with permission from BMJ Publishing Group Ltd.

**Online Supplementary Figure 2: Efficacy of abatacept (ATTAIN study) [7]**


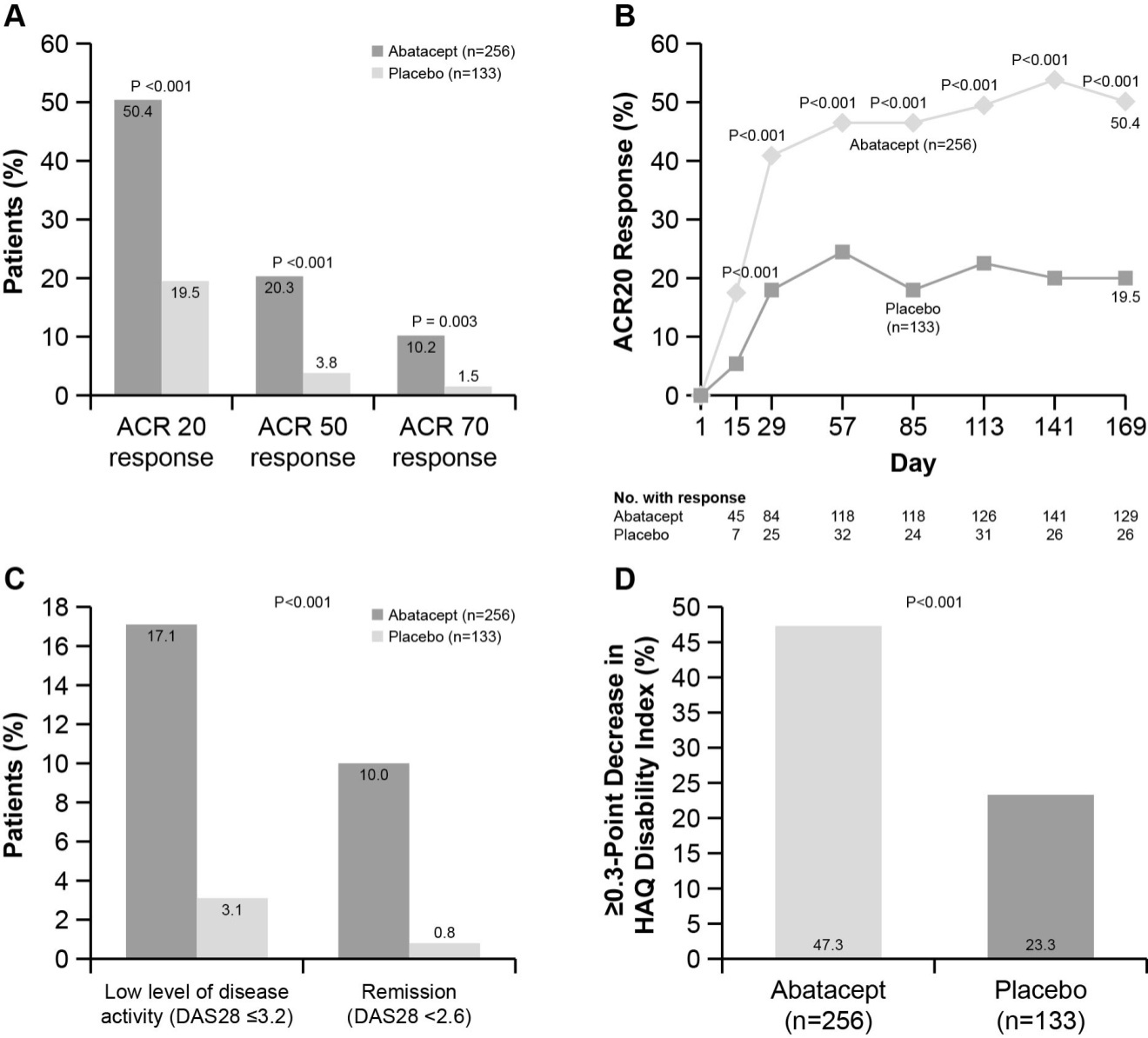


Clinical response to abatacept treatment by (A) rates of ACR 20, ACR 50, and ACR 70

responses at six months, (B) rates of ACR 20 responses at each post-baseline visit during the six-month study (numbers below graph are the numbers of patients with a response who were assessed), (C) rates of low levels of disease activity and remission at six months as defined with the use of the DAS28. Two patients in the abatacept group were excluded from the efficacy analysis because of a protocol violation; ACR=American College of Rheumatology; DAS=Disease Activity Score 28 **[Permission statement to be included upon receipt]**

**Online Supplementary References**

1. Emery P, Keystone E, Tony HP, Cantagrel A, van Vollenhoven R, Sanchez A, Alecock E, Lee J, Kremer J (2008) IL-6 receptor inhibition with tocilizumab improves treatment outcomes in patients with rheumatoid arthritis refractory to anti-tumour necrosis factor biologicals: results from a 24-week multicentre randomised placebo-controlled trial. Annals of the rheumatic diseases 67 (11):1516-1523

2. Kremer J, Fleischmann R, Brzezicki J, Ambs P, Alecock E, Burgos-Vargas R, Halland A (2009) Tocilizumab inhibits structural joint damage, improvoes physical function, and increases DAS28 remission rates in RA patients who respond inadequately to methotrexate: The LITHE study. Annals of the rheumatic diseases 68 ((Suppl13):122):Abstract OP-0157

3. Maini RN, Taylor PC, Szechinski J, Pavelka K, Broll J, Balint G, Emery P, Raemen F, Petersen J, Smolen J, Thomson D, Kishimoto T (2006) Double-blind randomized controlled clinical trial of the interleukin-6 receptor antagonist, tocilizumab, in European patients with rheumatoid arthritis who had an incomplete response to methotrexate. Arthritis Rheum 54 (9):2817-2829

4. Smolen JS, Beaulieu A, Rubbert-Roth A, Ramos-Remus C, Rovensky J, Alecock E, Woodworth T, Alten R (2008) Effect of interleukin-6 receptor inhibition with tocilizumab in patients with rheumatoid arthritis (OPTION study): a double-blind, placebo-controlled, randomised trial. Lancet 371 (9617):987-997

5. Genovese MC, McKay JD, Nasonov EL, Mysler EF, da Silva NA, Alecock E, Woodworth T, Gomez-Reino JJ (2008) Interleukin-6 receptor inhibition with tocilizumab reduces disease activity in rheumatoid arthritis with inadequate response to disease-modifying antirheumatic drugs: the tocilizumab in combination with traditional disease-modifying antirheumatic drug therapy study. Arthritis Rheum 58 (10):2968-2980

6. Jones G, Sebba A, Gu J, Lowenstein MB, Calvo A, Gomez-Reino JJ, Siri DA, Tomsic M, Alecock E, Woodworth T, Genovese MC Comparison of tocilizumab monotherapy versus methotrexate monotherapy in patients with moderate to severe rheumatoid arthritis: the AMBITION study. Annals of the rheumatic diseases 69 (1):88-96

7. Genovese MC, Becker JC, Schiff M, Luggen M, Sherrer Y, Kremer J, Birbara C, Box J, Natarajan K, Nuamah I, Li T, Aranda R, Hagerty DT, Dougados M (2005) Abatacept for rheumatoid arthritis refractory to tumor necrosis factor alpha inhibition. N Engl J Med 353 (11):1114-1123

8. Genovese M, Schiff M, Luggen M, Le Bars M, Becker JC, Aranda R, Li T, Elegbe M, Dougados M (2010) Safety, efficacy and health-related quality of life (HR-QoL) through 5 years of abatacept (ABA) treatmetn in patients with rheumatoid arthritis (RA) and an inadequate response to anti-tumour necrosis factor (TNF) therapy. Annals of the rheumatic diseases 69(Suppl3) (385):Abstract FRI0208

9. Schiff M, Pritchard C, Huffstutter JE, Rodriguez-Valverde V, Durez P, Zhou X, Li T, Bahrt K, Kelly S, Le Bars M, Genovese MC (2009) The 6-month safety and efficacy of abatacept in patients with rheumatoid arthritis who underwent a washout after anti-tumour necrosis factor therapy or were directly switched to abatacept: the ARRIVE trial. Annals of the rheumatic diseases 68 (11):1708-1714

10. Keystone E, Aranda R, Becker JC, Koncz T, Lafosse C, Le Bars M, Schmidely N, Genovese M (2008) Efficacy of abatacept through 1 year of the ATTAIN trial in patients with rheumatoid arthritis, regardless of reason for failure of prior anti-TNF therapy, or number of prior anti-TNF therapies used. Annals of the rheumatic diseases 67(Suppl II) (196):Abstract THU0187

11. Schiff M, Le Bars M, Gaillez C, Wu G, Poncet C, Genovese MC (2009) Efficacy and safety of abatacept in patients with rheumatoid arthritis and an inadequate response to anti-TNF therapy by number of prior anti-TNF therapies used. Annals of the rheumatic diseases 68(Suppl3):574.Abstract SAT0102

12. Kremer JM, Genant HK, Moreland LW, Russell AS, Emery P, Abud-Mendoza C, Szechinski J, Li T, Ge Z, Becker JC, Westhovens R (2006) Effects of abatacept in patients with methotrexate-resistant active rheumatoid arthritis: a randomized trial. Ann Intern Med 144 (12):865-876

13. Westhovens R, Kremer JM, Emery P, Russel AS, Li T, Aranda R, Becker JC, Zhao C, Dougados M (2009) Consistent safety and sustained improvement in disease activity and treatment response over 7 years of abatacept treatment in biologic-naive patients with RA. Annals of the rheumatic diseases 68 ((Suppl3):577):Abstract SAT0108

14. Hochberg M, Westhovens R, Aranda R, Kelly S, Khan N, Qi K, Pappu R, Delaet I, Luo A, Torbeyns A, Moreland L, Cohen R, Gujrathi S, Weinblatt M (2010) Lon-term safety profile of abatacept (ORENCIA) from the rheumatoid arthritis cliincal trial program; integrated analysis of data from 12,132 patient-years of exposure. Arthritis & Rheumatism 62 ((10 Suppl)):S164-165

15. Gottenberg JE, Ravaud P, Bardin T, Cacoub P, Cantagrel A, Combe B, Dougados M, Flipo RM, Godeau B, Guillevin L, Hachulla E, Le Loet X, Sibilia J, Schaeverbeke T, Barton P, Mariette X (2010) Prospective follow-up of abatacept treatment in 920 patients with refractory rheumatoid arthritis: tolerance and efficacdy data from the French ORA (ORENCIA and rheumatoid arthritis) registy Annals of the rheumatic diseases 69 ((Suppl3):386):Abstract FRI0211

16. Schiff M, Keiserman M, Codding C, Songcharoen S, Berman A, Nayiager S, Saldate C, Li T, Aranda R, Becker JC, Lin C, Cornet PL, Dougados M (2008) Efficacy and safety of abatacept or infliximab vs placebo in ATTEST: a phase III, multi-centre, randomised, double-blind, placebo-controlled study in patients with rheumatoid arthritis and an inadequate response to methotrexate. Annals of the rheumatic diseases 67 (8):1096-1103

17. Schiff M, Le Bars M, Reed DM, Becker JC, Aranda R, Vratsanos G, Zhao C, Dougados M (2009) Improvements in RA disease activity status in patients treated with abatacept versus infliximab: results from the ATTEST Trial Annals of the rheumatic diseases 68 ((Suppl3)741.):Poster AB0244
